# Nanoparticle-Encapsulated Camptothecin: Epigenetic Modulation in DNA Repair Mechanisms in Colon Cancer Cells

**DOI:** 10.3390/molecules26175414

**Published:** 2021-09-06

**Authors:** Aisha Farhana, Avin Ee-Hwan Koh, Jia Bei Tong, Abdullah Alsrhani, Suresh Kumar Subbiah, Pooi Ling Mok

**Affiliations:** 1 Department of Clinical Laboratory Sciences, College of Applied Medical Sciences, Jouf University, Sakaka 72388, Aljouf Province, Saudi Arabia; afalserhani@ju.edu.sa (A.A.); pooi_ling@upm.edu.my (P.L.M.); 2 Department of Biomedical Sciences, Faculty of Medicine and Health Sciences, Universiti Putra Malaysia, Seri Kembangan 43400, Selangor, Malaysia; avin.keh@gmail.com; 3 Department of Medical Microbiology, Universiti Putra Malaysia, Seri Kembangan 43400, Selangor, Malaysia; hanfabin1@gmail.com; 4 Centre for Materials Engineering and Regenerative Medicine, Bharath Institute of Higher Education and Research, Bharath University, Chennai 600073, Tamil Nadu, India

**Keywords:** DNA repair, epigenetic modulation, colon cancer, nanoparticles, transcriptome analysis

## Abstract

Molecular crosstalk between the cellular epigenome and genome converge as a synergistic driver of oncogenic transformations. Besides other pathways, epigenetic regulatory circuits exert their effect towards cancer progression through the induction of DNA repair deficiencies. We explored this mechanism using a camptothecin encapsulated in β-cyclodextrin–EDTA–Fe_3_O_4_ nanoparticles (CPT-CEF)-treated HT29 cells model. We previously demonstrated that CPT-CEF treatment of HT29 cells effectively induces apoptosis and cell cycle arrest, stalling cancer progression. A comparative transcriptome analysis of CPT-CEF-treated versus untreated HT29 cells indicated that genes controlling mismatch repair, base excision repair, and homologues recombination were downregulated in these cancer cells. Our study demonstrated that treatment with CPT-CEF alleviated this repression. We observed that CPT-CEF exerts its effect by possibly affecting the DNA repair mechanism through epigenetic modulation involving genes of *HMGB1*, *APEX1*, and *POLE3*. Hence, we propose that CPT-CEF could be a DNA repair modulator that harnesses the cell’s epigenomic plasticity to amend DNA repair deficiencies in cancer cells.

## 1. Introduction

Epigenetic mechanisms are essential for the ontogenesis of mammals and the maintenance of tissue-specific gene expression [1,2]. Response to developmental and environmental signals within the tissue can alter the cell epigenetics and may lead to aberrancies in phenotype, oncogenic transformations, and cancer progression [1,3,4,5]. The epigenetic mechanisms are deregulated in many forms of cancer including colorectal cancer [6], bladder cancer [7], and leukemia [8].

Research progress in cancer epigenetics has shown that epigenetic mechanisms in cancer undergo extensive reprogramming, including DNA methylation, histone modification, nucleosome localization, and non-coding RNA [2,9]. Two of the most common mechanisms are DNA methylation and histone modifications. DNA methylation is a process involving the addition of methyl groups to DNA regions, which typically leads to the repression of gene transcription. The two common types present in tumors are demethylation and de novo methylation of CpG islands [10]. These result in expression profiles that promote tumor growth. Histone modification is a form of post-translational modification occurring on histone proteins that regulate chromatin. In cancer, the deregulation of this process affects the maintenance of repressive chromatin, thereby causing an aberrant promotion of gene expression [11]. Genetic changes are irreversible, while epigenetic modifications are reversible. This characteristic makes epigenetic modifications a perfect target for therapeutic intervention for cancer [12].

Camptothecin (CPT) has been demonstrated to be an effective anti-cancer drug for various cancers. Due to its low solubility in aqueous media and active lactone ring instability at physiological pH [13,14], encapsulation by CEF (cyclodextrin–EDTA–FE_3_O_4_) can effectively increase its stability and feasibility for cancer therapy [15]. Encapsulation by nanomaterials is being widely researched as an effective means of drug delivery for cancer therapy [16]. One example is the use of lipid nanocapsules that allows efficient delivery of loaded drugs to the tumor tissue [17]. Cyclodextrin was used for the encapsulation in this study as it can act as a multifunctional core for diverse conjugation of drugs and other molecules [18]. Meanwhile, the superparamagnetic iron oxide nanoparticle (Fe_3_O_4_) possesses high microwave absorbance; hence, it can be used to track and kill the remnant of tumor cells [19].

In the previous study, we used camptothecin encapsulated in β-cyclodextrin–EDTA–Fe_3_O_4_ nanoparticles (CPT-CEF) for treating human colon cancer HT29 cell lines [15]. We found that CPT-CEF could induce cell apoptosis and growth inhibition by arresting the cell cycle and activating the mitochondrial apoptotic pathways. In normal cells, excessive DNA damage that cannot be repaired by DNA repair factors typically leads to cell death as a safety mechanism to prevent cancer [20]. However, tumor cells have altered or defective DNA-repair mechanisms that prevent them from undergoing apoptosis, which can be exploited therapeutically for developing new anti-cancer drugs [21,22]. As such, we would like to further examine whether genes involved in epigenetic control for DNA-repair mechanisms were affected during treatment with CPT-CEF. DNA damage and deficiency in immediate repair is the primary underlying cause for the switch from normal to malignant cells [23]. To achieve the study objective, we carried out comparative transcriptomic studies between treated and untreated cancer cells. We then attempted to profile the transcriptomics based on well-known databases such as Gene Ontology (GO), which is a repository for annotated gene interactions, and Kyoto Encyclopedia of Genes and Genomes (KEGG), which is a resource containing known biological pathways. Our results reveal three important genes, *HMGB1*, *APEX1*, and *POLE3,* that function in the epigenetic control of a DNA-repair mechanism, which modulates cancer in HT29 cells, and induce apoptosis.

## 2. Results and Discussion

### 2.1. Mapping of List of Differentially Expressed Genes (DEGs) to Its Gene Ontology Terms

Camptothecin demonstrates a broad-spectrum anti-cancer activity. The molecular target for camptothecin has been firmly established to be the human DNA topoisomerase-I [24]. In vivo experiments have shown that camptothecin has an inhibitory effect on tumor growth, especially on digestive tract tumors, leukemia, and bladder cancer [25,26,27,28]. The application of camptothecin in cancer treatment is limited due to its poor water solubility. However, modification with nanotechnology makes it feasible to be used in cancer therapy [29]. 

**Figure 1:**
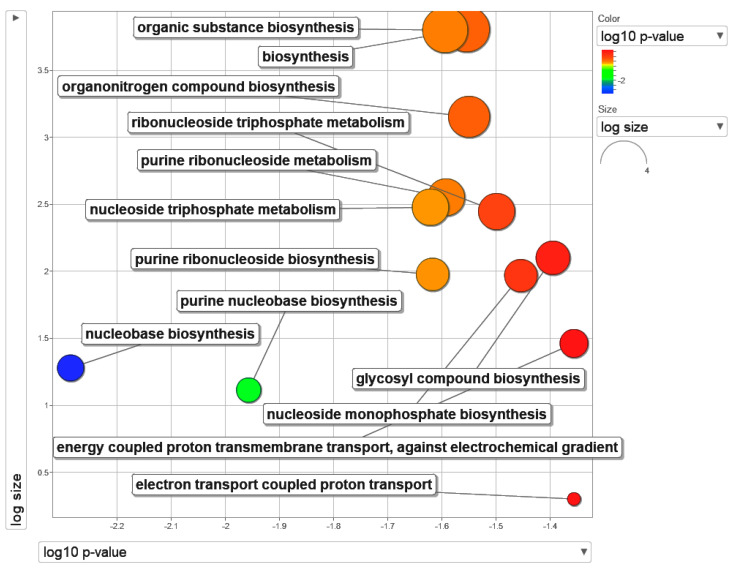
Visualization of the top terms in Gene Ontology (GO) biological processes obtained using over-representation analysis. The top GO terms were obtained using g:Profiler and clustered by ReVigo for visualization (adj *p* < 0.05). The data were plotted as log10 *p*-value against log size on a scatterplot. The GO terms were sized and colored according to the indicated scale.

In the present study, upon completion of sequencing, the data were processed to generate a list of DEGs. An ORA analysis was then performed using DEGs with the cut-off *p* < 0.05 and 2-fold changes. The ORA analysis generated results based on GO biological processes. REViGO was then used to summarize the terms (Figure 1). The top 13 enriched gene sets under GO biological processes were tabulated in Table 1. A majority of the terms involved nucleotide metabolism. In the previous study, we encapsulated camptothecin in β-cyclodextrin–EDTA–Fe_3_O_4_ nanoparticles (CPT-CEF) to enhance the solubility and stability, and further tested its inhibitory activity on HT29 cell growth in vitro. Our data shows CPT-CEF can induce caspase-3 activity and alter mitochondrial membrane potential, leading to arrest in the cell cycle, and ultimately apoptosis [15]. In addition, CPT-CEF treatment results in an enrichment of pathways involved in nucleotide metabolism (Figure 1).

### 2.2. Identification of Enriched KEGG Pathways in CPT-CEF-Treated HT29 Colon Cancer Cells

**Figure 2:**
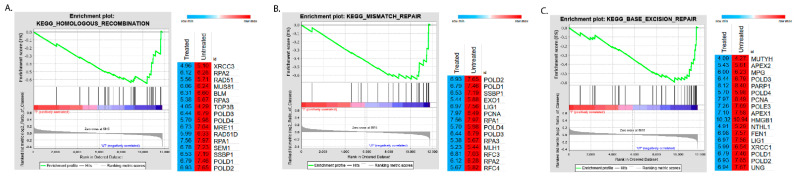
A gene set enrichment analysis (GSEA) of CPT-CEF-treated colon cancer cells revealed an enrichment of DNA repair mechanisms. The GSEA tool was used to make an enrichment plot of the homologous recombination (**A**), mismatch repair (**B**), and base excision repair (**C**) pathways. The genes that contributed to the leading-edge subset in the ranked list were shown in heatmaps, where the values indicated the mean normalized count data for each gene between CPF-CEF-treated and untreated samples. Red represents upregulation; blue represents downregulation.

In order to identify enrichment of pathways in either the treated or untreated control groups, a GSEA analysis was performed. KEGG was used as the reference database. The top 13 KEGG pathways were tabulated in Table 2. Similar to ORA analysis, a majority of the KEGG pathways involved played a role in nucleotide metabolism. There were also pathways involving DNA repair mechanisms. Our analysis showed that these pathways were enriched in the control samples. This suggests that CPT-CEF has an effect on processes involving nucleotide metabolism (Figure 2). The plots of these pathways are shown in Figure 2. The details are tabulated in Table 1 and Table 2, respectively. These pathways were enriched in the untreated control group, suggesting that they were unperturbed in that group. The genes from the leading edge of the data are tabulated in Table 3. Hence, we further hypothesized that CPT-CEF might modulate the DNA repair mechanisms by inducing changes in the epigenome of colon cancer cells. Disruption to DNA repair responses is one of the leading causes of the development of cancer cells [23].

It is worth noting that DNA repair mechanisms are also the responsible culprit for tumoral cell resistance to many cancer therapies [30]. Hence, in this study, we performed comparative transcriptomic analysis between CPT-CEF-treated and untreated HT29 cells to identify the dysregulated genes that are involved in signaling pathways associated with DNA repairs at the epigenetics level. The results of our study demonstrate that cancer cells downregulated a total of 47 genes controlling mismatch repair, base excision repair, and homologous recombination (Table 3).

### 2.3. Identification of Genes Involved in Epigenetic Modulation 

**Figure 3:**
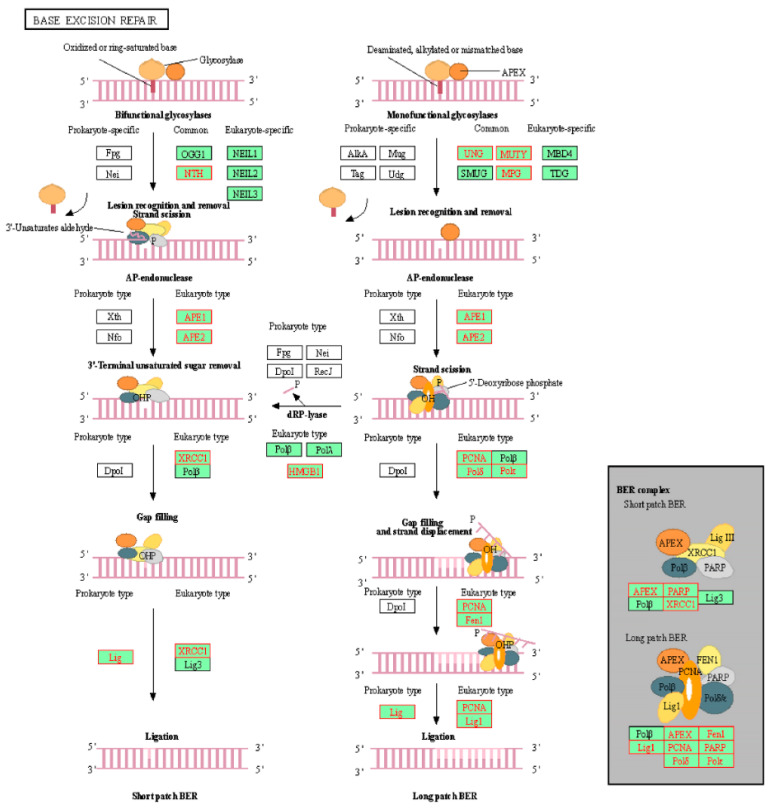
Visualization of the GSEA-enriched genes from the leading-edge subset of the base excision repair pathway in CPT-CEF-treated colon cancer cells. The pathway was extracted from the KEGG database, and the GSEA-enriched genes obtained from the data set were highlighted in red. The underexpression of these genes is predicted to have an impact in the downstream signaling involved during base excision repair.

**Figure 4:**
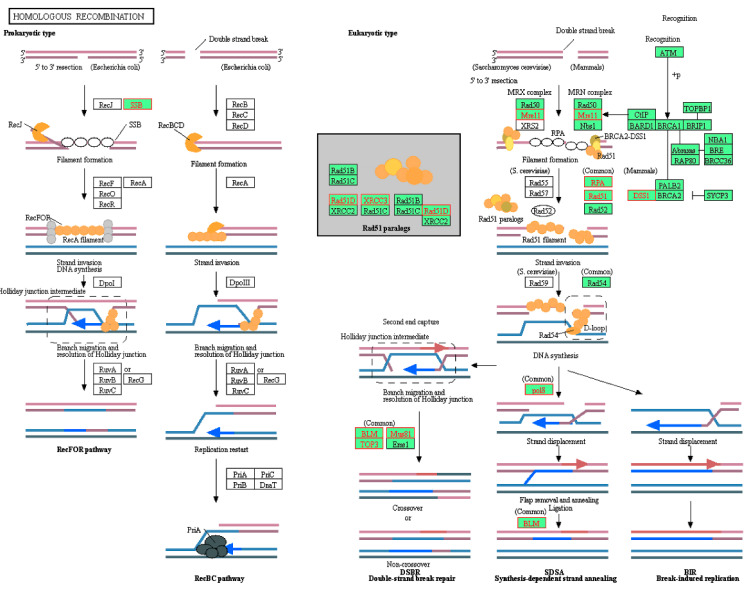
Visualization of the GSEA-enriched genes from the leading-edge subset of the homologous recombination pathway in CPT-CEF-treated colon cancer cells. The pathway was extracted from the KEGG database, and the GSEA-enriched genes obtained from the data set were highlighted red. The underexpression of these genes is predicted to have an impact in the downstream signaling involved during homologous recombination.

**Figure 5:**
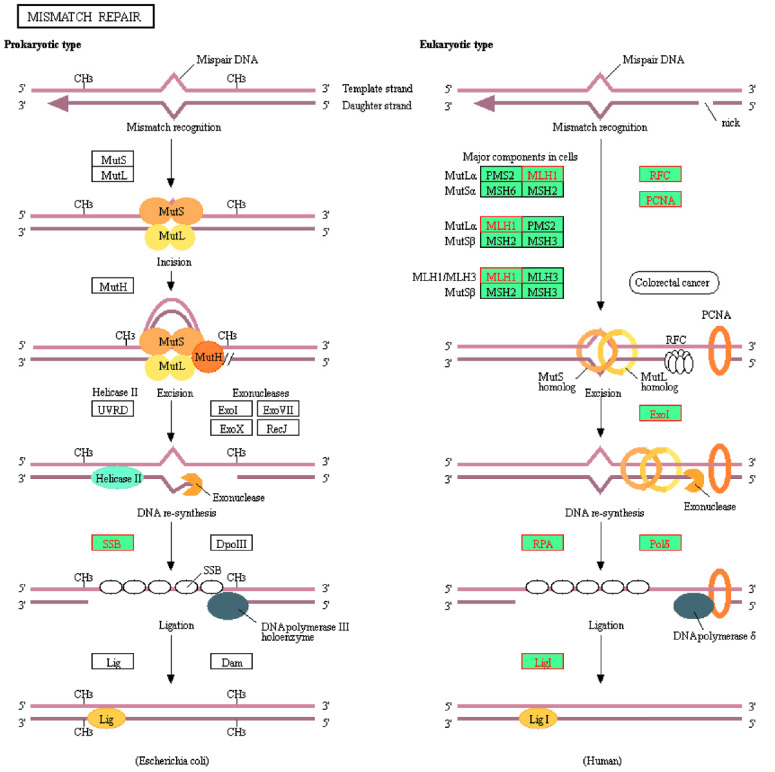
Visualization of the GSEA-enriched genes from the leading-edge subset of the mismatch repair pathway in CPT-CEF-treated colon cancer cells. The pathway was extracted from the KEGG database, and the GSEA-enriched genes obtained from the data set were highlighted red. The underexpression of these genes is predicted to have an impact in the downstream signaling involved during mismatch repair.

Genes identified in Table 4 were then cross-referenced with the epigenetics database. It was found that *HMGB1*, *APEX1*, and *POLE3* were involved with epigenetic modulation. These genes were members of the KEGG base excision repair pathway. As such, the position of these genes was visualized on the pathway (Figure 3). *POLE3* can be found as a subunit of DNA polymerase epsilon (Polε) on the KEGG map. Additionally, the homologous recombination and mismatch repair pathways were also visualized (Figure 4 and Figure 5, respectively). In the context of epigenetic study of cancer cell development, the analysis indicated that treatment with CPT-CEF could reverse these deficiencies by modulating the expression of these genes. To our knowledge, this is a novel study that successfully demonstrates the epigenome molecular interplay involved in DNA repair mechanisms when cancer cells are given anti-cancer drug therapy.

High mobility group box 1 (*HMGB1*) is a highly conserved expressed nuclear protein vital to reverse DNA damage and maintain genomic stability by preserving nucleosome structure and regulating DNA replication and transcription in cells [31]. Reduced activity of *HMGB1* leads to higher frequency of DNA damage when exposed to radiation, carcinogens, or chemotherapeutic or oxidative stress-inducing agents [32]. It can translocate from the nucleus to the cytoplasm following post-translational modifications, including acetylation, phosphorylation, and methylation [33]. The secretion of *HMGB1* from cells can trigger a cascade of inflammatory reactions. It can bind to Receptor for Advanced Glycation End products (RAGE) or Toll-like receptors (TLRs), which eventually activates the nuclear transcription factor κB (NF-κB) signal transduction pathway, and upregulates various apoptotic factors to cause cell death [31,33]. Meanwhile, *APEX1*-encoded DNA-(apurinic or apyrimidinic site) lyase has been shown to be highly expressed in a variety of tumors, including liver [34], colorectal [35], gastric [36], and non-small-cell lung cancer [37]. Gene expression profiling demonstrated that increased APE1 correlated with glioblastoma recurrence in patients [38]. The result of Gene Set Enrichment Analysis (GSEA) on hepatocellular carcinoma showed that the expression of *APEX1* was related to a DNA damage repair pathway and its high expression could result in poor prognosis [39]. *POLE3* is one of the four components in the DNA polymerase epsilon holoenzyme [40]. *POLE3* can interact with other histone-fold proteins to bind to DNA in a sequence-independent manner and initiate the synthesis of the leading strand during replication of DNA [41]. Mutations in *Polε* are associated with colon and endometrial cancer, and hence downregulation of *POLE3* could interrupt DNA repair [42].

In addition, this study also found that PKM gene expression was modified upon treatment with CPT-CEF in the HT29 cell line. Pyruvate Kinase Muscle (PKM) is a group of isozymes made up of *PKM1* and *PKM2*. PKM has direct control of the transcription activity of genes related to various cells’ metabolism. Therefore, aberrancy in PKM expression could lead to tumorigenesis [43,44,45]. Prior studies have shown that *PKM2* has a close relationship with cellular proliferation, migration, anchorage-independent growth, and in tumor growth and liver metastasis in vivo [46,47,48,49]. *PKM2* was demonstrated to promote dsDNA breaks repair through homologous recombination, and has been suggested as a cause of cancer resistance to genotoxic therapies [50]. We can corroborate that the repression of *PKM2* may affect the histone machinery through epigenetic alterations.

In a bigger context, aberrant epigenetic modification of these genes could lead to deregulated signal transduction as well as a promotion of tumorigenic processes. For example, the suppression of *HMGB1* can lead to a loss of maintenance in genomic stability, and as such, the regulation of DNA replication and transcription would be affected, which leads to uncontrolled cell proliferation. On the other hand, *POLE3* functions in DNA extension, and its aberrant downregulation could affect DNA repair, thereby leading to more mutations and further tumor progression. As such, further investigations into these candidates could yield newer formulations that can effectively target these epigenetic mechanisms to impede tumor growth.

## 3. Materials and Methods

### 3.1. Treatment of HT29 Colon Cancer Cells with CPT-CEF Nanocompound

A stock of the CPT-CEF nanocompound solution was prepared by dissolving it in 10% dimethyl sulfoxide (DMSO) (Nacalai, Kyoto, Kyoto, Japan) in complete culture medium before the experiment. The HT29 human colorectal carcinoma cell line used in this study was obtained from the Laboratory of Vaccine and Immunotherapy (LIVES) Institute of Biosciences (IBS, Seri Kembangan, Selangor, Malaysia), UPM. Firstly, the cells were seeded at a concentration of 1 × 10^4^ cells/mL of culture medium in a 6-well culture plate. It consisted of the Roswell Park Memorial Institute (RPMI) 1640 Medium (Nacalai, Kyoto, Kyoto, Japan), 10% Fetal Bovine Serum (Nacalai, Kyoto, Kyoto, Japan) and 1% Penicillin/Streptomycin. The culture was then incubated at 37 °C in a 5% CO_2_ incubator for 24 h. After that, the supernatant was discarded. The nanocompound solution was then added to the culture medium to obtain a final concentration of 133.5 μg/mL (IC50) (*n* = 3). The DMSO content in the stock solution did not exceed 1% of final nanocompound concentration in the culture medium. The cell culture was then incubated for 48 h in a 5% CO_2_ incubator before conducting the RNA-seq experiment.

### 3.2. Isolation of Total RNA from HT29 Colon Cancer Cells and Library Preparation for Sequencing

The HT29 colon cancer cell line was incubated with CPT-CEF, as mentioned in our previous study [15]. Then, the total RNA was extracted from the cells using the RNeasy mini kit according to the recommendation from the manufacturer (Qiagen, Hilden, Germany). Samples with an RNA integrity (RIN) value of more than 7 and high purity (A260/A280 ratio = 2) were used for the downstream experiment. The Bioanalyzer 2100 system (Agilent Technologies, Santa Clara, CA, USA) was used to measure RNA integrity, whereas the NanoDrop 2000 Spectrophotometer (Thermo Scientific, Waltham, MA, USA) was used to measure the A260/A280 ratio.

### 3.3. Library Preparation for RNA Sequencing

Library preparation was performed using the NEBNext^®^ Ultra™ II RNA Library Prep Kit for Illumina^®^ (New England Biolabs, Ipswich, MA, USA) according to the manufacturer’s protocol. The isolated RNA was further enriched to obtain mRNA. Total RNA was incubated with 50 µL of oligo dT beads and binding buffer at 65 °C for 5 min in a PCR tube. The tubes were then placed onto a magnetic stand to capture the beads. The contents of the tubes were washed several times to remove residual rRNA and other RNA types. Then, the beads were mixed with 50 µL of Tris buffer and incubated in a thermal cycler at 80 °C for 2 min, followed by a cooling step to a temperature of 25 °C before elution.

The mRNA was used to synthesize the first-strand cDNA. A reaction mix was set up and incubated for 10 min at 25 °C, 15 min at 42 °C, and 15 min at 70 °C, before finally putting it on hold at 4 °C. Immediately after that, the samples were incubated with the reaction mix for 1 h at 16 °C to produce the second cDNA strand. Purification of the double-stranded cDNA was then carried out using NEBNext Sample Purification Beads, and magnetic capture of the DNA strands was carried out. cDNA was eluted in Tris-EDTA buffer and used to ligate to unique adaptors to create cDNA libraries by incubating at 20 °C for 15 min. The ligation reaction was then purified using the NEBNext Sample Purification Beads. Finally, PCR enrichment of adapter-ligated cDNA was performed to expand the library before sequencing. The quality of the library was then assessed using the Bioanalyzer 2100 system. All the samples showing a single peak with the size of approximately 300 bp on the electropherogram were used for RNA-seq.

### 3.4. RNA-Seq Data Processing and Differential Gene Expression Analysis

The Illumina HiSeq2000 system (Illumina, Hayward, CA, USA) was used for the RNA-seq study. Paired-end sequencing was performed (2 × 100 bp). Once completed, the quality of the sequences was assessed using the FastQC tool. Alignment, annotation, and quantification were then performed using the Salmon tool (available at github.com, accessed 13 January 2021) and the GRCh38 homosapiens assembly (asia.ensembl.org, accessed 13 January 2021) as a reference transcriptome. Differential expression analysis of CPT-CEF-treated and untreated colon cancer cells were then performed using the DeSeq2 tool (Bioconductor.org, accessed 14 January 2021). After performing the analysis, the differentially expressed genes were extracted.

### 3.5. Functional Enrichment Analysis and Identification of Genes in Epigenetics

The g:Profiler tool was used to perform over-representation analysis (ORA) (https://biit.cs.ut.ee/gprofiler/, accessed 16 January 2021). By using a threshold of *p* < 0.10 and 2-fold change, the DEGs were loaded into the tool. Gene Ontology (GO) was the database used for the study. The output files were summarized and clustered using REViGO (http://revigo.irb.hr/, accessed 16 January 2021). Functional enrichment was carried out using the GSEA tool (https://www.gsea-msigdb.org/gsea/index.jsp, accessed 16 January 2021). KEGG (c2.cp.kegg.v7.2.symbols.gmt, accessed 16 January 2021) was the database used for this analysis. The data were annotated using the database from MSigdb (Human_ENSEMBL_Gene_ID_MSigDB.v7.2.chip, accessed 16 January 2021). The default settings were used for the analysis, except for the following parameters: permutation number—1000; permutation type—gene set; gene ranking metric—log2 ratio of classes. Data mining was then performed by cross-referencing the enriched gene sets with the EpiFactors database (https://epifactors.autosome.ru/, accessed 16 January 2021). The associated genes in epigenetics were extracted and tabulated.

## 4. Conclusions

The defect of DNA repair mechanisms in human colon cancer might be due to the downregulated genes that have a significant role in controlling the transcription of genes involved in base excision repair. Overall, this study postulates that treatment with CPT-CEF could inhibit the proliferation of HT29 cells, most probably by reversing this dysregulation. Current transcriptomic results deserve our attention as many pieces of literature have pointed out that efficient DNA repair in cancer cells could confer resistance to radio- and chemotherapy. More mechanistic studies have to be performed to confirm the results and to re-evaluate the role of these genes before formulating a strategy to prevent or halt the cancer cell growth.

## Figures and Tables

**Table 1:** A list of the top 13 gene ontology terms for biological processes that was obtained by over-representation analysis using g:Profiler (adj *p* < 0.05).

GO Term ID	Description	Log_10_ Adj *p*
GO:0046112	Nucleobase biosynthetic process	−2.2861
GO:0015988	Energy-coupled proton transmembrane transport, against electrochemical gradient	−1.3562
GO:0009058	Biosynthetic process	−1.5541
GO:1901576	Organic substance biosynthetic process	−1.5941
GO:1901566	Organonitrogen compound biosynthetic process	−1.55
GO:0009199	Ribonucleoside triphosphate metabolic process	−1.499
GO:0015990	Electron transport coupled proton transport	−1.3562
GO:0009124	Nucleoside monophosphate biosynthetic process	−1.4542
GO:0009141	Nucleoside triphosphate metabolic process	−1.6213
GO:0046128	Purine ribonucleoside metabolic process	−1.5925
GO:1901659	Glycosyl compound biosynthetic process	−1.3951
GO:0046129	Purine ribonucleoside biosynthetic process	−1.6173
GO:0009113	Purine nucleobase biosynthetic process	−1.9572

**Table 2:** A list of the top 13 KEGG pathways found enriched in CPT-CEF-treated colon cancer cells. Gene set enrichment analysis was performed using the KEGG database as a reference (adj *p* < 0.05). NES, normalized enrichment score.

WikiPathways ID	NES	Adj *p* Value
KEGG_RIBOSOME	−2.61902	0
KEGG_DNA_REPLICATION	−2.4385	0
KEGG_PURINE_METABOLISM	−2.24033	0
KEGG_PYRIMIDINE_METABOLISM	−2.20893	2.31 × 10^−4^
KEGG_SPLICEOSOME	−2.13383	5.56 × 10^−4^
KEGG_FRUCTOSE_AND_MANNOSE_METABOLISM	−2.10173	0.001798
KEGG_HOMOLOGOUS_RECOMBINATION	−2.09315	0.001541
KEGG_GLYCOLYSIS_GLUCONEOGENESIS	−2.07396	0.001349
KEGG_MISMATCH_REPAIR	−2.04734	0.001544
KEGG_BASE_EXCISION_REPAIR	−2.04304	0.001389
KEGG_CYSTEINE_AND_METHIONINE_METABOLISM	−2.01921	0.001616
KEGG_ONE_CARBON_POOL_BY_FOLATE	−1.99589	0.002156
KEGG_PROTEASOME	−1.95564	0.002531
KEGG_PENTOSE_PHOSPHATE_PATHWAY	−1.92024	0.003016

**Table 3:** A list of GSEA-enriched genes from CPT-CEF-treated colon cancer cells that are involved in the DNA repair mechanism. The genes from the leading-edge subset were ranked using GSEA and tabulated. KEGG was used as the reference map. RMS stands for ranked metric score; RES stands for running enrichment score. The genes were annotated based on Ensembl database.

Pathway	Symbol	Description	RMS	RES
Homologous recombination	RPA2	replication protein A2	−0.03701	−0.60918
	RAD51	RAD51 recombinase	−0.04012	−0.59841
	XRCC3	X-ray repair cross complementing 3	−0.04147	−0.57749
	MUS81	MUS81 structure-specific endonuclease subunit	−0.04274	−0.55398
	BLM	BLM RecQ-like helicase	−0.06435	−0.60186
	MRE11	MRE11 homolog, double strand break repair nuclease	−0.06466	−0.55804
	POLD4	DNA polymerase delta 4, accessory subunit	−0.0695	−0.52524
	RPA3	replication protein A3	−0.0747	−0.48659
	RPA1	replication protein A1	−0.07625	−0.43647
	POLD3	DNA polymerase delta 3, accessory subunit	−0.07674	−0.38431
	RAD51D	RAD51 paralog D	−0.08042	−0.33793
	TOP3B	DNA topoisomerase III beta	−0.08197	−0.28399
	SEM1	SEM1 26S proteasome complex subunit	−0.0914	−0.2371
	POLD1	DNA polymerase delta 1, catalytic subunit	−0.13575	−0.18254
	SSBP1	single-stranded DNA-binding protein 1	−0.13701	−0.08714
	POLD2	DNA polymerase delta 2, accessory subunit	−0.14252	0.009967
Mismatch repair	RPA2	replication protein A2	−0.03701	−0.6119
	RFC4	replication factor C subunit 4	−0.03721	−0.58618
	RFC3	replication factor C subunit 3	−0.0476	−0.60649
	MLH1	mutL homolog 1	−0.0573	−0.60656
	POLD4	DNA polymerase delta 4, accessory subunit	−0.0695	−0.59973
	RPA3	replication protein A3	−0.0747	−0.55899
	RPA1	replication protein A1	−0.07625	−0.50674
	POLD3	DNA polymerase delta 3, accessory subunit	−0.07674	−0.45243
	PCNA	proliferating cell nuclear antigen	−0.09206	−0.41689
	EXO1	exonuclease 1	−0.11252	−0.35987
	LIG1	DNA ligase 1	−0.11726	−0.27812
	POLD1	DNA polymerase delta 1, catalytic subunit	−0.13575	−0.19036
	SSBP1	single-stranded DNA-binding protein 1	−0.13701	−0.09113
	POLD2	DNA polymerase delta 2, accessory subunit	−0.14252	0.009963
Base excision repair	APEX2	apurinic/apyrimidinic endodeoxyribonuclease 2	−0.04908	−0.5695
	PARP1	poly(ADP-ribose) polymerase 1	−0.04922	−0.54643
	MPG	*N*-methylpurine DNA glycosylase	−0.05495	−0.54497
	MUTYH	mutY DNA glycosylase	−0.06143	−0.54262
	POLD4	DNA polymerase delta 4, accessory subunit	−0.0695	−0.53485
	POLD3	DNA polymerase delta 3, accessory subunit	−0.07674	−0.51601
	POLE3	DNA polymerase epsilon 3, accessory subunit	−0.08351	−0.49272
	HMGB1	high mobility group box 1	−0.08388	−0.45306
	PCNA	proliferating cell nuclear antigen	−0.09206	−0.42234
	APEX1	apurinic/apyrimidinic endodeoxyribonuclease 1	−0.09397	−0.37987
	NTHL1	nth-like DNA glycosylase 1	−0.10643	−0.34445
	LIG1	DNA ligase 1	−0.11726	−0.29743
	FEN1	flap structure-specific endonuclease 1	−0.11774	−0.24157
	XRCC1	X-ray repair cross complementing 1	−0.12694	−0.18614
	POLD1	DNA polymerase delta 1, catalytic subunit	−0.13575	−0.12503
	POLD2	DNA polymerase delta 2, accessory subunit	−0.14252	−0.05904
	UNG	uracil DNA glycosylase	−0.14332	0.00946

**Table 4:** Several genes involved in gene expression regulation were also found to be involved in epigenetic modifications. The genes were data-mined and annotated using EpiFactors as a reference database. FC, fold change. NA indicates data not available.

Symbol	Description	Function	Target Molecule	Target Entity	Product	Comment	Adj *p*	log_2_FC
PKM	pyruvate kinase, muscle	Histone modification write cofactor	histone	H3S10, H3S28, H2BS32	H3S10ph, H3S28ph, H2BS32ph, H3T11ph	Transcriptional activation by epidermal growth factor (EGF) is mediated via phosphorylation of H3S10 H3S28 and H2BS32 by Rsk-2 and PKM2.	1.02 × 10^−7^	−1.02522
HMGB1	high mobility group box 1	Chromatin remodeling	chromatin	NA	NA	Chromatin-specific remodeling by HMGB1 and linker histone H1 silences proinflammatory genes during endotoxin tolerance.	0.01295	−0.64422
APEX1	APEX nuclease (multifunctional DNA repair enzyme) 1	DNA modification cofactor	DNA	NA	NA	UniProt: May play a role in the epigenetic regulation of gene expression by participating in DNA demethylation.	0.075289	−0.63047
POLE3	polymerase (DNA directed), epsilon 3, accessory subunit	Histone chaperone	histone	NA	NA	The human homologues of two novel putative histone-fold proteins in Drosophila CHRAC are present in HuCHRAC. The two human histone-fold proteins form a stable complex that binds naked DNA but not nucleosomes.	0.109001	−0.54009

## Data Availability

The datasets presented in this study can be found in the Gene Expression Ominibus (GEO) public repository with accession number GSE165875.

## Sample Availability

Samples of the compounds are not available from the authors.
